# Round the Hospitals

**Published:** 1921-06-18

**Authors:** 


### ROUND THE HOSPITALS.

No less a sum than £21,300 has been realised up to date from the nine days' scenic fair at Birmingham on behalf of the Birmingham and Three Counties Centre of the College of Nursing, and for the establishment of a club for all trained nurses in the three counties. Such a result is a remarkable tribute to the esteem in which nurses are held in this great Midland centre, and a triumphant proof

**ROUND THE HOSPITALS**—(*continued*).

of what may be accomplished by energy and efficient work on the part of the organisers of such a festival. We offer our congratulations to all so concerned. The last day of the fair was as brilliant as its predecessors, and Lady de Frece (Vesta Tilley) in declaring the bazaar opened presented a donation of £100.

From the nursing point of view the Royal Sanitary Institute Congress at Folkestone from June 20 to 25 will be of considerable interest. On the morning of Tuesday, June 21, Miss E. M. Wyatt will read a paper on the need of further co-operation between health visitors and district nursing associations, and Miss Hannah Weir will read a paper on the trained nurse in public health work. Both these subjects offer a wide field for discussion; and though they have been dealt with time and again, there are many points still to be brought to the surface and threshed out. We hope, therefore, that all interested in public health work, and particularly trained nurses, will make it their business to be present. Further information as to the hour and place of the meeting may be obtained from one of the honorary local secretaries of the Congress at the Town Clerk's Office, Folkestone. Both Miss Wyatt and Miss Weir, who are reading the papers mentioned above, are trained nurses and members of the College of Nursing Health Advisory Committee.

Next week is a very busy one. Following on the Folkestone Congress is the annual meeting at Edinburgh, on June 24, of the College of Nursing. On the evening of the same day there will be a Conference in the Surgeons' Hall, Nicholson Street, Edinburgh, at 8 p.m., to which all trained nurses and nurses in training are invited. Mrs. George Ker, LL.D., J.P., will be in the chair, and papers will be read on (1) "The Nurse in Relation to the Development and Practice of Scientific Medicine," by Dr. J. C. Meakins, Professor of Therapeutics, Edinburgh University; (2) "The Controversy Concerning the Trained Nurses' Suitability for Public-health Work," by Miss Watt, Superintendent of Health Visitors and Queen's Nurses, Borough of Motherwell and Wishaw; (3) "The Status of the Nursing Profession from an Economic Point of View," by Miss May Dunbar, Trained Nurse, Secretary to the Scottish Women's Friendly Society. The perusal of the list of these subjects must make all who can decide to travel to Edinburgh, from wherever they may be, and listen to or take part in discussions on subjects which embrace the whole field of nursing and its relation to the public wellbeing.

The entrance examination for the five sister-tutor studentships (two College, two Cowdray, and one London Centre) will be held on Saturday, June 18, at 10 a.m. and 2 p.m., simultaneously at the College of Ambulance, 56 Queen Anne Street, London, 8 Drumsheugh Gardens, Edinburgh, and the Mater Infirmorum Hospital, Belfast. Between thirty and forty candidates have entered. To provide the £150 required for the London Centre studentship a special fund is being raised, to which many members of the Centre promised donations; these have not all materialised yet, and the Secretary, we understand, is anxiously awaiting them.

It is very satisfactory to find that an effort has been made in preparing the schedule for the Census next Sunday to induce "nurses" to state whether their calling be "nurse (domestic)," "monthly nurse," or "sick nurse," etc. If this be conscientiously carried out we shall have at last the means of forming a reliable estimate as to the numerical strength of the nursing profession. It is much to be desired also that married nurses not following their profession should describe themselves as belonging to it and "retired." An immense fund of potential nursing ability is stored away among married nurses, as we discovered when war broke out, and if they are content merely to describe their occupation as "none" or "home duties" the opportunity of finding out the complete total of nurses will be lost.

The Ministry of Pensions announce that nurses disabled by service in the Great War who intend to claim alternative retired pay or pension, based on pre-war earnings and present earning capacity, must make application before July 2, 1921, or before a year has elapsed since the first award of disability retired pay or pension, whichever is the later date. Under the terms of the Royal Warrant a disabled nurse is not eligible for alternative pension if her pre-war earnings did not exceed £95 a year. As the substitution of alternative retired pay or pension for the ordinary flat-rate pension represents in some cases a substantial addition to the amount of the pension, disabled nurses who have not yet made application should ascertain without loss of time whether they are entitled to the benefits of the alternative system. Applications and inquiries should be addressed to the Officers' Branch, Ministry of Pensions, Cromwell House Annexe, Millbank, S.W. 1.

The chief lady superintendent of Lady Minto's Indian Nursing Association is Miss R. E. Darbyshire, R.R.C., and under her "sympathetic and able administration," to quote the words of the Hon. Secretary, Colonel Heard, I.M.S., in the lately issued report, and her enthusiasm in the cause of nursing in India, a splendid year's work has been accomplished. In 1920 all previous records were surpassed, over 100 more patients having been nursed than before. Thirty-three sisters went out, of whom twenty-six were new members, while seven rejoined. Twenty-six sisters left the Association, twelve to be married and seven on the expiration of their contract; two were invalided and five released at their own request. In many places, particularly in Calcutta, the housing difficulty retards an expansion urgently called for. The number of nursing homes in charge of sisters is increasing, and

**ROUND THE HOSPITALS—(*continued*).**

from almost all the centres comes the report that more sisters could have been employed if available. The cost of passages is the largest item in the expenditure, and Lord Inchcape's generous subscription of £1,000 for three years on behalf of the P. and O. Company is most timely.

From Nyasaland comes the news that the work of the hospitals is in full swing once more. Dr. Wigan, medical officer to the Universities' Mission to Central Africa (headquarters 9 Dartmouth Street, S.W. 1), reports instead of three nurses there are now seven nurses actually at work, with a hope of one or two new arrivals and two nurses on furlough. There is urgent need for further subscribers, and, in spite of increased cost, the sum of three guineas is sufficient to provide food, mat, and blanket for one patient for a year.

The Overseas Nursing Association reports a crowded year's work, having very largely developed its sphere since the war. Whereas the highest number of nurses sent out previously was 96 in 1914, the numbers totalled 144 in 1920, with 118 already in the current year. The total number working in connection with the Association is 431. The new developments include a home sister in Singapore, three additional nursing sisters in Nyasaland, fifteen in the Federated Malay States, and one at Johore Bahru; two private nurses at Kuala Lumpur. The matron sent last year to the Colonial Hospital at Fiji has been reinforced by two sisters. Private nurses have been sent to Madrid and are going out to Guatemala. In British Columbia and Newfoundland Association nurses are taking up work. The names of four sisters have been added to the roll of merit—viz., Miss Pallott, R.R.C.; Mrs. Thomas; Miss M. A. Lee; and Miss Nevill, R.R.C., who was killed in a motor accident in Nigeria, where she had been working since 1899.

It would be thought that there would be a run on the Sea-Bathing Infirmary, Margate, in response to their advertisement for staff nurses at a salary of £60 to £65. These are just such appointments as ought surely to attract newly certificated nurses whose training has taken place in London or other large city. The admirably equipped wards, with their broad, open-air balconies, look out over the sea in such close proximity to it that at high tide there is almost the impression of being on board ship, minus attendant drawbacks. The work is exhilarating, for cures are the order of the day, and it opens up a prospect of future success in the lucrative work of a public tuberculosis nurse. Will our readers mention it to their friends? The probationers' course is well-suited for girls making their first plunge into institution life.

The contributions to the cause of higher education freely made by the leading voluntary hospital training-schools are very little known to the general public, and have been hitherto entirely ignored by grant-making authorities. In reality the process of training nurses has become a vast and far-reaching educational system. In many respects it is unique. In its effects on character it is probably unrivalled. It is extremely costly, and has been evolved on such free lines that it has passed almost insensibly through widely varied experimental stages to an established standard recognised as the highest so far obtainable. Among the recommendations proffered by Viscount Cave's Committee to the Ministry of Health, we observe with much satisfaction one which urges the payment from funds available for technical education of grants for the training of nurses. Much encouragement would be given to modern methods of teaching if such grants were to take the form of subsidising sister-tutors whose services from the purely educational point of view are now recognised as indispensable.

The Steyning Guardians have been informed by the Clerk to the Ministry of Health that the Minister cannot properly intervene in the case of the two nurses whose certificates the medical officer has declined to sign. The matter is again left to the decision of the Guardians, and as the examiner will not sign the certificates it appears to the Ministry, as to the Guardians themselves, that they are helpless in the matter. The nurses were admitted at the last meeting of the Guardians and permitted to state their case, but no further action was taken. We suggest that the matron of some other institution with vacancies on her staff be asked to give the nurses a chance of retrieving their *faux pas*. It is possible that under different conditions they may yet do good service. We note with satisfaction that their names have not been given to the press.

In the effort to turn nursing into a "trade," and procure for nurses the supposititious advantages enjoyed by apprentices, the extraordinary contention has arisen that probationers who contract for three years' service are not liable to dismissal. It is quite true that a nurse instantly dismissed from one hospital does not often find a post in another. It would be deplorable if she did. Summary dismissal is a very rare occurrence. It can only be justified by convincing evidence furnished to those acquainted with the nurse's work and conduct that she is a danger, directly or indirectly, to the sick under her care. A probationer who is recklessly negligent in carrying out orders, deliberately rough and unkind to her patients, or guilty of inciting others to insubordination is as dangerous in an institution full of sick people as an apprentice who persisted in using a naked light would be in a coal mine. In neither case is delay tolerable, beyond the time necessary for investigation of the circumstances.

Gold, silver and bronze medals for meritorious services were presented to probationers from the North-Eastern, North-Western and Park Hospitals on June 4, at the meeting of the Metropolitan Asylums Board, by the chairman. Reference was made to the splendid organisation of the nursing staff during the epidemic of scarlet fever last year, when 1,300 more patients than ever before were treated at the hospitals under the board.

